# Modulatory effects of mesenchymal stem cells on microglia in ischemic stroke

**DOI:** 10.3389/fneur.2022.1073958

**Published:** 2023-01-18

**Authors:** Lei Hao, Yongtao Yang, Xiaoli Xu, Xiuming Guo, Qunling Zhan

**Affiliations:** ^1^Department of Neurology, The First Branch of The First Affiliated Hospital of Chongqing Medical University, Chongqing, China; ^2^Department of Neurology, The First Affiliated Hospital of Chongqing Medical University, Chongqing, China; ^3^Department of Neurology, The Fifth People's Hospital of Chongqing, Chongqing, China

**Keywords:** mesenchymal stem cell, extracellular vesicles, microglia, ischemic stroke, cell polarization

## Abstract

Ischemic stroke accounts for 70–80% of all stroke cases. Immunity plays an important role in the pathophysiology of ischemic stroke. Microglia are the first line of defense in the central nervous system. Microglial functions are largely dependent on their pro-inflammatory (M1-like) or anti-inflammatory (M2-like) phenotype. Modulating neuroinflammation *via* targeting microglia polarization toward anti-inflammatory phenotype might be a novel treatment for ischemic stroke. Mesenchymal stem cells (MSC) and MSC-derived extracellular vesicles (MSC-EVs) have been demonstrated to modulate microglia activation and phenotype polarization. In this review, we summarize the physiological characteristics and functions of microglia in the healthy brain, the activation and polarization of microglia in stroke brain, the effects of MSC/MSC-EVs on the activation of MSC *in vitro* and *in vivo*, and possible underlying mechanisms, providing evidence for a possible novel therapeutics for the treatment of ischemic stroke.

## Introduction

Stroke is a major disease leading to long-term disability and high near-term mortality in the world. Ischemic stroke caused by arterial occlusion accounts for 70–80% of all stroke cases, and intracerebral hemorrhage (ICH) is responsible for 10–20% (1). Currently, intravenous (IV) thrombolysis with tissue-type plasminogen activator (t-PA) and endovascular thrombectomy constitute the standard treatments for acute ischemic strokes. However, only 3.4 and 5.2% of all patients with acute ischemic stroke (AIS) are suitable for the treatments due to the extremely narrow therapeutic time window limit (2). Most patients with stroke, who are not eligible for the treatments, are still subjected to neurological deficits such as sensory/motor and/or cognitive impairment (3). To date, there is still no effective treatment to improve stroke patient outcomes in the subacute to chronic phases (4). There is an urgent need for developing efficacious therapies for ischemic stroke.

Immunity has been demonstrated to play a key role in the pathophysiology of ischemic stroke. Modulation of immune responses after ischemic stroke provides novel therapeutic strategies for patients who are not fit for IV thrombolysis and endovascular thrombectomy (5). As the resident immune cells in the central nervous system (CNS), microglia continuously monitor the brain tissue and are one of the first cells to respond to brain injury. The resident microglia are rapidly activated and alter their phenotypes and functions when responding to changes in the local CNS microenvironment after cerebral ischemia. These cells exert either a pro- (M1-like) or anti-inflammatory (M2-like) role according to distinct phenotypes. M1-like microglia aggravate brain damage (6, 7), whereas M2-like microglia enhance brain restoration (8, 9). Both M1 and M2-like microglia have high plasticity and are capable of shifting to other phenotypes according to the cerebral environment (10). Therefore, modulating neuroinflammation *via* targeting the polarization of microglia to a neuroprotective phenotype might be suitable for developing novel therapeutics for stroke.

Many preclinical studies demonstrated the immunomodulatory properties of MSC in ischemic stroke (11–14). It has been demonstrated that MSC secretion-based paracrine effects are the main mechanism of MSC-mediated therapeutic effects rather than direct cellular replacement (15). MSC may exert their functions in a paracrine manner related to the production of extracellular vesicles (EVs). EVs are membranous particles originating from numerous types of cells, which are enriched with various proteins, lipids, and nucleic acids and act as important mediators of intercellular communication (16). Therapeutic use of MSC-EVs has been attractive in recent years due to their several advantages, including lower risk of immunogenicity and microvascular thrombosis, easy crossing to the blood–brain barrier, easy high production of genetically modified EVs, and great ability to transfer active particles to a target due to a higher surface to volume ratio (17).

MSC mainly exerts immunosuppressive effects on the immune system. It has been demonstrated that MSC can suppress the proliferation, activation, and secretion of pro-inflammatory cytokines of various immune cells such as NK cells, macrophages, and T and B cells (18–21). Mounting evidence demonstrated that MSC and MSC-EVs may improve neurological function by modulating microglial activation and polarization in stroke. In this review, we summarize the effects of MSC and MSC-EVs on microglia in ischemic stroke.

## Definition of microglia

Microglia originate from erythromyeloid progenitors in the yolk sac. Microglia precursor cells migrate to the brain and differentiate into microglia during embryological development, of which cardinal functions include synaptic remodeling and maturation (22). In adults, microglia are widely distributed across the whole brain as the resident immune cells of the CNS. The main function of microglia is to maintain brain homeostasis by constantly surveying the CNS microenvironment. Microglia are rapidly activated and migrate to the lesion area after ischemic stroke, which is also called microgliosis. Resident microglia are the main source of microgliosis (23). A study showed that the local residual microglia rather than microglia progenitors mainly contribute to the microglia proliferation (24). Ischemic stroke can promote rapid and complex changes in microglial morphologies and functions.

## Morphology of microglia in the healthy and stroke brain

In the brain of healthy adults, the density of microglia is regionally specific. There are more microglia in the hippocampus and the olfactory bulb and fewer microglia in the fiber bundle (25). Resting ramified microglia, associated with immune surveillance and maintenance of homeostasis, have much more long, thin, and highly branched mobile processes under physiological conditions. When stimulated by environmental changes, ramified microglia are transformed to be amoeboid with an enlarged cell body and shorter processes (26). Morphological transformation is usually accompanied by functional responses such as migration, antigen presentation, and phagocytosis (27). There are various morphologies of microglia in different brain regions (28). For example, compared with white matter, microglia in the gray matter have smaller somas and process extensions. Microglia produce effects on CNS surveillance *via* constantly extending and retracting their branches (29). When microglia cells migrate from gray matter to white matter, their morphology may change from ramified to amoeboid shape with rounded cell bodies and processes along the direction of the fibers (30, 31). The number and morphology of microglia are also different in different regions such as the cortex, the amygdala, the hippocampus, and the preoptic area (POA) between sexes (32–35). Phagocytic microglia are significantly more in the hippocampus of female rats than in male rats 2–3 days after birth (33). Fewer microglia were observed in the female rat pup POA than male at postnatal 2 days (36). Neonatal males had much more activated ameboid microglia in POA than females (36). Microglia from female rats had smaller cell bodies, more process branch points, and greater process length than males (36). Adult male mice had a higher density of Iba+ microglia in the hippocampus, cortex, and amygdala than female mice (32).

Microglia will transform into a more spherical shape and coupe with toxic stimuli when exposed to challenges in the microenvironment (29, 37). Microglial cells can exhibit four types of distinct morphology, namely, ramified, intermediate, amoeboid, and round, when activated after stroke (38). Ramified microglia represent the resting state mainly stay distally to the ischemic core or in the contralateral hemisphere, while intermediate microglia have larger cell bodies and shorter bumps. Conversely, amoeba microglia have larger bodies and shorter processes, or even no processes, similar to round microglia in the lesion center (5, 38). The total volume of reactive microglia gradually increased and reached a peak on the 4^th^ day after ischemic stroke (23). In the first few days, only sparse reactive microglia were found in the ischemic core region. In the later stage, more reactive microglia accumulate in the core zone. The density of microglia with a hypertrophic cell body and no ramified processes was the highest in the accumulation zone surrounding the ischemic core. Reactive microglia in the marginal zone between the accumulation zone and outside the non-ischemic area exhibited fewer short and stout processes than resting microglia (23). In another study, Iba1-positive microglia showed different morphological appearances in the control and the ischemic hemisphere (39). Ramified microglia with long thin processes mostly occurred in the normal area, whereas intermediate microglia with swollen processes were localized in the infarction border zone. Amoeboid microglia with round shape and no processes were mostly observed in the ischemic core between 3 and 7 days after stroke (39).

### The function of microglia in the healthy and stroke brain

Microglia actively and continuously monitor the cerebral microenvironment by extending and retracting their processes in adult brain (40). The main role of microglia in a healthy brain is a spectrum of surveilling functions, including normal phagocytosis and synaptic pruning. When stimulated by cell injury, microglia rapidly activate and shift to pro- or anti-inflammatory phenotype. Both spatial and temporal dimensions are very important in microglia dynamics because microglia present with pro- or anti-inflammatory phenotype depending on specific pathophysiological stages and brain regions after stroke (41). Pro-inflammatory microglia (M1-like) secrete detrimental cytokines and molecules such as interleukin (IL)-6, IL-1β, NO, TNF-α, IL-12, and IL-23 and express surface markers such as CD16 and CD32, which aggravate brain injury. However, anti-inflammatory microglia (M2-like) secrete anti-inflammatory cytokines such as IL-4, IL-10, transforming growth factor-β (TGF-β), IL-13, and growth factors such as vascular endothelial growth factor (VEGF) and brain-derived neurotrophic factor (BDNF) and express markers such as CD206,arginase1 (Arg1), TGF-β, insulin growth factor 1 (IGF-1), and IL-10, which promote neurological recovery (40, 42, 43). The M2-like microglia are further categorized as M2a, M2b, and M2c depending on their cellular function (5, 44, 45). M2a microglia, induced by IL-13 or IL-4, are involved in tissue regeneration and repair. M2a microglia also produce significant amounts of Arg1, Ym-1, CD206, and Fizz1 (5, 45). Microglia of M2b phenotype, activated by immune complexes such as Fcγ receptors, Toll-like receptors (TLRs), or IL-1R, exert immunoregulatory effects (5, 44, 46). The M2c phenotype microglia, deactivated by IL-10, TGF-β, and glucocorticoids, promote tissue regeneration when the inflammation reaction recedes (5, 47, 48). The function of microglia is also different between sexes. Most genes associated with inflammatory processes, including regulation of cell migration and cytokine production, are more expressed in male mice microglia compared with females (49). Male microglia are more prone to inflammatory reactions than female microglia due to transcriptionally activated NF-κB (49). However, female microglia have much more expression of genes related to cell plasticity, inflammatory response control, and repair than male microglia (49).

In a transient ischemic stroke model, activated Iba1^+^microglia appeared within 24 h and reached the peak within 4–7 days in the infarct core area. However, in the peri-infarct region, microglia emerged much earlier within ~3.5 h and peaked at 7 days (50). The temporal kinetics of microglia/macrophage polarization was described in a mouse model of middle cerebral artery occlusion (MCAO) (10). Microglia/macrophages initially attracted to the ischemic area exhibit mainly the M2 phenotype (CD206^+^Iba^+^ double-positive cells) only for a short period of time within 7 days post ischemia. The early recruitment of M2-like microglia/macrophages may act to clean ischemic tissue and restrict brain damage. M1-like microglia/macrophages (CD16/32^+^Iba^+^ double-positive cells) with decreased phagocytosis and enhanced secretion of pro-inflammatory mediators increasingly occurred from day 3 and remained high level until day 14 after ischemia. These cells may exacerbate neuronal demise and impair axon regrowth. Hence, the “helpful” M2-like microglia were first induced in response to ischemic injury during the early, acute phase of ischemic stroke, of which the function is protecting the neurons in the infarct area, phagocytizing cellular debris, and helping to restrict the area of damage. Then, the “harmful” M1-like microglia become more abundant in the next several days, and the neuronal damage caused by ischemia is increased. The shift of M2- to M1-like microglia in the process of chronic inflammation after stroke exacerbate the neuronal injury, leading to reduced neuronal recovery.

Microglia may respond differently between sexes under ischemic stroke. When subjected to pMCAO, male mice exhibited larger ischemic lesions than female mice (49). The progression of damage was significantly lower in male mice after transplantation of female microglia, indicative of the protective effects of female microglia (49). This phenomenon can be probably interpreted by the fact that Ym1 (a marker for microglia anti-inflammatory activation) immunoreactivity in Iba1-positive cells surrounding the ischemic lesion was higher in male mice transplanted with female microglia than in control (49). Hence, sex-specific microglia phenotype is intrinsically independent of the hormonal environment.

With the development of recent technology such as single-cell RNA sequencing and cytometry by time-of-flight mass spectrometry (CyTOF), spatial, temporal, and functional diversity of microglia during development, homeostasis, and disease in mice and humans has been unveiled (51). Varying degrees of pro- and anti-inflammatory markers may coexist on the same microglial cell. Hence, microglia may exhibit multiple phenotypical subtypes *in vivo* rather than two individual states based on the clustering of transcriptomic data. For example, 14 microglia sub-clusters were discovered in the cortex penumbra at the early stage of ischemic stroke without complete expression of M1 or M2-type marker genes (52). Five distinct microglial subtypes were identified in the mouse model of MCAO, which did not have a complete overlap with classic M1 or M2 subsets at the single-cell level (53). At least six transcriptionally distinct microglial subsets were uncovered in the stroke-aged brain (54). Further research on temporal and regional heterogeneity of activated microglia based on transcriptomic studies will be helpful in better understanding the pathophysiological mechanism of ischemic stroke.

### Effects of MSC/MSC-EVs on morphological changes of microglia

MSCs, a population of non-hematopoietic cells, were identified for the first time in the bone marrow (55). Subsequently, MSCs have been successfully isolated and cultured from various tissues in mammals, including circulating blood, umbilical cord blood (UCB), menstrual blood, the placenta, the heart, the adipose tissue, the skeletal muscle, the pancreas, and the dental pulp (56, 57). MSC surface markers include CD105, CD73, and CD90, and more than 95% of the culture do not express CD34, CD45, and CD14 or CD11b, CD79a or CD19, and HLA-DR (58).

MSCs of different origins have been demonstrated to exert immunomodulatory effects on microglia. One study investigated the effects of adipose tissue-derived mesenchymal stem cells (AMSCs) on microglia morphology and involved intracellular molecules *in vitro* (59). The results showed that AMSCs were capable of inducing a round, flat microglia phenotype (inflammatory) into a ramifying, anti-inflammatory one. Ramified microglia induced by AMSC have decreased secretion of the pro-inflammatory cytokines such as TNF-a and IL-6, increased phagocytic activity, and the upregulation of neurotrophic factors and of the Arg-1 (a marker for M2-like regulatory microglia) (59). AMSC-secreted CSF-1 binds to its receptor, resulting in phosphorylation of PI3K and ERK1/2, followed by activation of small RhoGTPases, actin polymerization, and cell morphology change and migration (59). The ratio of ramified to hypertrophic microglia was significantly increased in the ischemic brain at 12 weeks when MSC-EVs were IV administered in monkeys 24 h and 14 days after ischemic injury (60). Moreover, microglia in the perilesional gray (PG) of MSC-EV-treated monkeys showed significantly greater branching complexity, process intersections, and length compared with control (60). Therefore, MSC-EV treatment could shift microglial morphology from hypertrophic to ramified microglia, which present with stronger surveillance capacity and homeostatic functions (60).

### MSC/MSC-EVs modulating microglia activation and phenotype shift *in vitro*

MSC transplantation is neuroprotective after stroke at least in part *via* modulating microglia-mediated neuroinflammation. MSC exhibited inhibition of the pro-inflammatory cytokine expression, such as TNF-α, IL-1β, IL-6, and IFNγ, pro-inflammatory enzyme expression, such as iNOS and COX-2, and chemokine expression, such as MCP1 and GRO-α, when co-cultured with LPS, IL-β, or IFNγ-primed HMO6 or BV2 microglia (61–63) (Table 1). However, MSC-produced paracrine factors may account for its therapeutic effect, considering that >99% of transplanted MSC has been entrapped in the lungs. A study on MSC secretome showed that EVs/exosome was the only component that could successfully reproduce most of the beneficial effects exerted by parent cells (71). *In vitro*, oxygen- and glucose-deprived (OGD) model can activate microglial-mediated inflammatory response and simulate the ischemic/reperfusion microenvironment *in vivo*. Several *in vitro* studies demonstrated that exosomes from MSC could promote M1 to M2 microglia shift in OGD conditions (Table 1). Notably, hypoxia preconditioning could increase the production of exosomes by bone marrow MSC (BMSC), whereas inhibiting the secretion of exosomes to a great extent eliminated its effects on M1 to M2 microglia shift in OGD (66). Therefore, exosomes play pivotal roles in the beneficial effects of MSC on microglia. As a marker of MSC-Exos, miRNAs have been considered the most important component and nearly can reproduce most of the exosomal effects on recipient cells (72). MicroRNA (miR) are small, endogenous, non-coding RNA molecules that can selectively hybridize to the 3′ untranslated region (UTR) poly(A) tail of targeted mRNAs, leading to the prevention of their transcription into proteins or promotion of their degradation (73). When co-cultured with primary microglia cells under OGD conditions, exosomes from adipose-derived stem cells (ADSCs) overexpressing miR-30d-5p have a greater effect in inhibiting inflammatory factor expression and promoting M2 microglial shift by inhibiting autophagy (64). MiR-126^+^ exosomes can also inhibit microglial activation and the expression of inflammatory factors such as TNF-α and IL-1β in the OGD model (65). Human umbilical cord mesenchymal stem cells (hUCMSC)- derived exosomal miR-26b-5p could inhibit M1 polarization of microglia *via* targeting CH25H to suppress the TLR pathway (68). Rat BMSC-derived exosomal miR-223-3p induced conversion of inflammatory M1 microglia toward M2 microglia *via* downregulating CysLT2R transcription and expression (67). hUCMSC-exosomal miR-145-5p was demonstrated to attenuate OGD-induced microglial pro-inflammatory activity, evidenced by lowered expression of IL-6, TNF-α, and IL-1β, *via* suppression of the IRAK1/TRAF6 signaling pathway (70). Compared with EVs derived from other MSC sources, EVs from the umbilical cord have the ability to exert stronger therapeutic immunomodulation and protective effects (74).

**Table 1:** MSC modulating microglial activation and polarization *in vitro*.

**MSC/MSC-EVs**	**Microglia**	**Effect**	**Mechanism**
hMSC line, B10	IL-1β or IFNγ stimulated human microglia cell line, HMO6	Reduced expression of iNOS, Cox-2 and MCP-1 in HMO6 cells	IL-5 and fractalkine (61).
Mouse BMSC	LPS stimulated BV2 cell model	Reduced expression of expression of TNF-α, IL-1β, IL-6, and iNOS.	Attenuated activation of NF-κB signaling and the phosphorylation of p38, JNK, and Erk via TSG-6 (62).
AMSCs	LPS stimulated BV2 cells	Decreased pro-inflammatory cytokines, including IFN-γ, IL-1β, TNF-α, and IL-6, chemokines MCP1 and GRO-α, and nitrite accumulation increased CD200 and TGF-β	CD200 expression on AMSC (63).
Rat ADSC exosome	Primary rat microglia exposed to OGD	Decreased number of Iba+iNOS+ microglia and increased number of Iba+CD206+ microglia	Suppression of autophagy *via* miR-30d-5p (64).
Rat ADSC exosome	BV2 cells exposed to OGD	Reduced production of TNF α and IL-1β	miR126 (65)
Rat BMSC-CM	BV2 microglia exposed to OGD	Decreased pro-inflammatory cytokines including TNF- α, IL-1β and IL-6. Increased anti-inflammatory cytokines IL-10, CD206 and Arg-1	Secretion of exosomes (66).
Rat BMSC exosome o/e MiR-223-3p	BV2 cells exposed to OGD and cysteinyl leukotrienes (CysLTs) stimulation	Decreased CD16/32+cells, increased CD206+cells, decreased pro-inflammatory factors (IL-6 and IL-1β) and increased anti-inflammatory factor (IL-10)	Through inhibiting CysLT2R mediated signaling pathway (67).
hUCMSC-exsome o/e miR-26b-5p	Mouse microglia exposed to OGD	Decreased CD11b+iNOS+ M1 cells and increased CD11b+Arg + M2 cells. Decreased inflammatory factors such as TNF-α, IL-6 and CCL2	By targeting CH25H to inactivate the TLR pathway (68).
Rat BMSC exsome	BV2 cells exposed to OGD	Lower expression of M1 markers including CD32, CD68 and iNOS, higher expression of M2 markers including CD206, Arg and Ym1.	(69)
hUMSC exosome	BV2 cells exposed to OGD	lowered expression of IL-6, TNF-α, and IL-1β	miR-146a-5p suppression of the IRAK1/TRAF6 signaling pathway (70).

BV2, murine microglial cells; OGD, oxygen and glucose deprivation; o/e, overexpressing.

### MSC/MSC-EVs modulating microglia activation and phenotype shift *in vivo*

A pile of evidence demonstrated the immunomodulatory effects of MSC or MSC-EVs on microglia in cerebral ischemia model (Table 2). The most commonly used species include rat and mice, whereas monkey was used in one study (60). MSC or MSC-EVs were administrated mainly *via* IV route, and others include intra-arterial, intracerebral, and intranasal routes. When stem cells were infused in stroke models, most of the animal studies have chosen the acute (within 48 h) and subacute (within 7 days after ischemia) phases as the transplantation time. In one study, hUCMSC was administered IV 2 weeks before cerebral ischemia (87). The number of M1-like microglia (Iba^+^CD16/32^+^cells) decreased and M2-like microglia (Iba^+^CD206^+^cells) increased 7 days after photothrombotic stroke (87). These results indicated that MSC could also be developed as a prophylactic therapy for patients post-stroke besides therapeutic treatment. Most preclinical studies showed that the number of activated microglia was significantly decreased by MSC or EV administration (15, 61, 63–65, 75, 77–82, 84, 85, 88). Notably, three-dimensional (3D) spheroid cultured MSCs have been demonstrated to exhibit decreased cell size; therefore, these cells are not likely to be entrapped in lung tissue after IV infusion (39). Moreover, the increased expression of chemokine receptor CXCR4 after 3D culture enhanced the homing ability of the MSC to the ischemic brain area. The number of amoeboid microglia decreased significantly 5 days after MCAO. The mRNA expression of microglial markers Iba1 was reduced. 3D-cultured MSC downregulate pro-inflammatory mediators, including IL-12β, Hmgb1, Cxcl10, Ccl2, and IL-1β and increase expression of anti-inflammatory mediators STC1, HGF, and TSG-6 probably *via* decrease of Mincle expression in microglia cells (39). 3D-cultured MSC has been shown to have enhanced anti-inflammatory effects on microglia, which provide a novel and efficient method for the treatment of immune-mediated disorders. Exosomes derived from MSC of different origins have been demonstrated to exert enhancing effects on microglia polarization from the M1 to M2 phenotype. For example, ADSC-derived exosomes can reduce the number of M1-like microglia (Iba1^+^/iNOS^+^cells) and increase the number of M2-like microglia (Iba1^+^/CD206^+^cells) at 3 days after MCAO (64). hUCMSCs-exos can also attenuate I/R-induced M1 polarization (68, 70). The number of M1-like microglia (Iba1^+^/CD32^+^ cells) was statistically decreased in MCAO rats treated with BMSC-Exos, while the number of M2-like (Iba1^+^/CD206^+^cells) microglia was significantly increased (69). Hence, IV-administered BMSC-Exos can shift microglia toward neuroprotective M2 phenotype in the subacute phase of ischemia stroke (69).

**Table 2:** Therapeutic use of MSC/MSC EVs in preclinical ischemic stroke animal models.

**Animal models**	**MSC/** **MSC EVs**	**Dose**	**Route**	**Time**	**Behavioral measure**	**Infarct volume**	**Outcomes**
Rat MCAO	Rat BMSC	3 × 10^6^	IV	7 d post ischemia	Adhesive-removal patch test mNSS	Amelioration	Reduced number of GSI-B4+microglia/macrophages 4 months after MCAO (75)
Rat MCAO	Rat BMSC hMPC 32F line	1 × 10^5^	IV	1 d or 7 d after stroke	Climbing test mNSS	Amelioration	Activated CD11+ microglia increased 10 d after cell infusion (76)
Rat MCAO	human MSC line, B10	3 × 10^6^	IV	1 d after MCAO	NA	NA	Decreased Iba-1+ cell accumulation at 3 and 7 d after MCAO *via* secreting Fractalkine and IL-5 (61).
Mouse MCAO	Mouse BMSC	1 × 10^6^	IV	24 h post ischemia	NA	NA	Reduced TGF-β1+IB4+ cells 14 d after MCAO (77).
Rat MCAO	human MSC	1 × 10^6^	IA	1 d, 4 d or 7 d after stroke	Cylinder test	Amelioration	Reduced infiltration of ED-1+ microglia (78)
Rat MCAO	human MSC line B10	3 × 10^6^	IV	24 h after stroke	mNSS	Amelioration	Decreased IL-1b, TNFa, iNOS, and MCP-1 in Iba+ cells by modulation of NF-κB pathway (79).
Rat MCAO	BMSC CM		IV	Immediately after blood reperfusion	Grasping power test	Amelioration	Reduced infiltration of ED-1+ micrglia/macrophage in the ischemic cortex (80)
Rat MCAO	AD-MSC	5 × 10^5^	IA	1 d after MCAO	Rotarod test mNSS	Amelioration	Reduced Iba+ cells at 1 or 4 wk after cell infusion (81)
Rat MCAO	AMSC	1 × 10^5^	IC	24 h after surgery	Rotarod motor test mNSS	Amelioration	Decreased Iba+ cells 1 wk after cell transplantation *via* CD200 expression on AMSC (63).
Rat MCAO	hUC-MSCs	1 × 10^6^ or 4 × 10^6^	IV	24 h after surgery	Inclined plane system adhesive removal test modified forelimb foot fault placing test mNSS	Amelioration	Attenuated accumulation of hypertrophic Iba-1-positive microglia cells in the ischemic brain regions (82).
Rat MCAO	rat BMSCs	1 × 10^6^	IV	1 h	Cylinder test and grid walking test	Amelioration	increased IGF-1+CD68+ and BDNF+Iba-1+ double positive cells (83).
Rat MCAO	Rat ADSC exsome	80 ug	IV	Immediately	NA	Amelioration	Reduced expression of the M1 marker, iNOS and increased expression of the M2 marker, CD206 at 3d after MCAO through miR-30d-5p suppressing autophagy (64).
Rat MCAO	hAD-MSC	1 × 10^6^	IV	48 h after surgery	The Rogers, beam walking, and adhesive removal tests	No difference	Reduced number of Iba-1-positive cells at 6w post stroke (84)
Monkey cortical injury	BMSC EV	4 × 10^11^ particles/kg	IV	24 h and 14 d after injury	Hand Dexterity Task	Amelioration	Increased density of ramified Iba1+/LN3+ microglia a higher ratio of ramified: hypertrophic phenotypes. Increased microglial ramification in PG but not SW at 12 wk (60).
Focal brain injury	hBMSC or EVs	5 × 10^5^ 1.3 × 10^9^	IA	48 h	NA	NA	Reduced infiltration of ED-1+ micrglia/macrophage (85).
Rat MCAO	Rat ADSC exsome		IV	2 h after MCAO	Foot-fault test mNSS	NA	Reduced Iba+ cells *via* miR-126 (65).
Rat MCAO	hUC-MSC exsome	100 ug	IV	1 d after MCAO	Morris water maze test mNSS	NA	Decreased CD16 and IL-1β and increased CD206 and Arg-1 expression at 4 d and 14 d; reduced number of CD16/iba-1 and increased number of CD206/iba-1 at 14 d (86).
Mouse Photothrombotic stroke model	hUC-MSC	~10^6^ hUC- MSCs/20 g	IV	2 week before ischemic stroke	NA	No difference	Decreased Iba+CD16/32+ and increased Iba+CD206+ microglia 7 days after stroke through down-regulation of H3 methylation (87).
Mouse MCAO	hUCMSC-exsome overexpressing miR-26b-5p	50 ug/ml	IV	immediately after ischemia	Longa scoring system	Amelioration	Decreased expression of iNOS and increased Arg1 24 h after reperfusion by targeting CH25H to inactivate the TLR pathway (68).
Rat MCAO	Rat BMSC	1 × 10^6^	IV	1 h post dMCAO	NA	NA	Reduced number of TGF-β1/Iba-1+ microglia Cells at 48 h after ischemia (88).
Mouse MCAO	hUC-MSC exsome	50 ug	IV	4 h post perfusion	Bederson scale and higher grip strength test	Amelioration	Decreased IBA-1+ CD16+ increased IBA-1+CD206+ microglia cells at 72 h post-stroke (70).
Rat MCAO	human AD-MSC	2 × 10^6^	IV	2 or 7 d after surgery	Cylinder test	No difference	Number, staining intensity, and morphological changes not be affected at 44 days after stroke (89).
Rat MCAO	MSC from human placenta	3 × 10^6^	IV	3 d after MCAO	Adhesive-removal test mNSS	Amelioration	Decreased number of amoeboid microglia 5 d after MCAO. Decreased Iba1 expression. Downregulation of pro-inflammatory IL-12β, Hmgb1, Cxcl10, Ccl2, and IL-1β, and increase of anti-inflammatory STC1, HGF, and TSG-6 *via* decrease of Mincle expression in microglia cells 5 d after MCAO (39)
MCAO in neonatal mice	Mouse BMSC sEV	5 ug	ICV or IN	After reperfusion	NA	Amelioration	Reduced Iba1+ surface area within the ischemic core, reduced level of IL-6,KC, MCP-1 and MIP1αat 72 after MCAO (15).
Rat MCAO	Rat BMSC exosome	80,100,120 ug	IV	2 h after MCAO	Brain water content, Morris water maze, and CatWalk system	Amelioration	Reduced number of M1 microglia (Iba1+/CD32+ cells), increased number of M2 microglia (Iba1+/CD206+ cells) in ischemia penumbra 24h after reperfusion (69).
Rat MCAO	Rat BMSC	1 × 10^5^	ICV	24 h after MCAO	Longa scoring system	Amelioration	Increased number of Iba+ CD206+ cells and reduced number of Iba+ TNFα+ cells in the infarct region especially at 3 d and 7 d post transplantation *via* CX3CL1 (90).

MCAO, middle cerebral artery occlusion; BM, bone marrow; MSC, mesenchymal stem cell; hMSC, human mesenchymal stem cell; AD-MSC, adipose-derived mesenchymal stem cell; AMSC, human placenta amniotic membrane-derived mesenchymal stem cells; hUC-MSC, human umbilical cord–derived mesenchymal stem cells; ADSC, adipose derived stem cells; PG, perilesional gray matter; SW, sublesional white matter; mNSS, modified neurological severity score; NA, not assessed; IV, intravenous; IA, intraarterial; IC, intracerebral; ICV, intracerebroventricular; IN, intranasal; BV2, murine microglial cells.

However, there were some conflicting results. Compared with 7 days post-ischemia, administration of either allogeneic (rMSCs) or xenogeneic (hMSCs) stem cells at 1 day resulted in a significantly greater recovery of motor behavior after MCAO that was possibly related to an increase in activated microglia (CD11) in the infarct region (76). Transplantation of BMSC reduces the proportion of Iba-1^+^microglia cells expressing TGF-β in the cerebral infarction area 48 h after ischemia (88). IV allogenic BMSC significantly increased the inflammation, evidenced by an increase of TNFαand IL-1βand the number of Iba+ microglia/macrophages in the ischemic core cortex at day 2 after MCAO (83). Hence, MSC delivered at 2 days after MCAO can stimulate but not suppress the immune response (83, 88). It is reasonable that the immunosuppressive capacity of MSC requires a certain amount of time to be induced under inflammatory conditions. The inflammatory reaction will be induced by MSC without being fully activated, followed by exacerbated disease outcome (83). Therefore, the immunosuppressive and anti-inflammatory effects of MSC may be dependent on time. The neurotrophic effects of microglia, evidenced by an increased number of IGF-1^+^CD68^+^ and BDNF^+^Iba-1^+^ double-positive cells, are most likely responsible for the therapeutic effects of MSC in the acute phase (83). However, in another study, ADMSCs delivered IV at 2 or 7 days post-cerebral ischemia do not have a profound influence on the number or phenotype of Iba+ microglia in the perilesional cortex at 44 days after pMCAO (89). This may be the reason that subpopulations of microglia with different phenotypes were recruited to the perilesion and were involved in tissue repair in different activation stages (89, 91). There are some factors that probably account for these controversial effects of transplanted cells on the number and activation of microglia, including the route, timing, and dose of transplantation, follow-up time, stroke model, and techniques of staining and counting (89).

As mentioned above, most of the *in vivo* studies did not demonstrate the real causal effects of MSC or MSC-EVs on microglia in stroke. It was possible that morphological and phenotype transformation of microglia *in vivo* may be secondary. Moreover, neuroprotective function of MSC on ischemic stroke is comprehensive. The effect of MSC on microglia cannot completely account for the amelioration of functional outcome in ischemic stroke. This is because MSC can also promote macrophage polarization toward anti-inflammatory phenotype (92–94). Besides, infiltration of immune cells such as monocytes and neutrophils to inflammation sites can be prevented by MSC (93, 95). Selective elimination of microglia subtype may help better understand the role of MSC in stroke. Most *in vivo* studies just observed morphological and phenotype changes of microglia after transplantation of MSC or MSC-EVs in stroke model. Accompanied functional alteration has not been explored in detail. Considering various dynamic states of microglia subtype may be present, determination of microglia by M1 or M2 phenotype based on surface markers is oversimplified and inappropriate. The function of numerous microglia subtype *in vivo* will be future research focus. Indeed, most of the *in vivo* studies even cannot reliably distinguish microglia from infiltrating macrophages just by detection of biomarkers. The two kinds of immune cells may be distinguished based on CCR2 expression (96). Combined with the extent of CD45 and CD11b expression, the resident microglia can be characterized as CD11bhi/CD45lo/CCR2^−^, and infiltrating macrophage as CD11blo/CD45hi/CCR2^+^ (97). Further research is needed for a precise identification of microglia and macrophage *in vivo*. Considering that EVs contain proteins, lipids, and nucleic acids, molecular profiles of these EVs will be the focus to determine which components may exert beneficial roles on microglia in ischemic stroke. Besides, the underlying specific molecular mechanisms of MSC in modulating microglia *in vivo* need to be fully elucidated.

## Mechanisms of MSC/MSC-EVs modulating the microglia activation and phenotype shift

### Transcription factors

**Figure 1:**
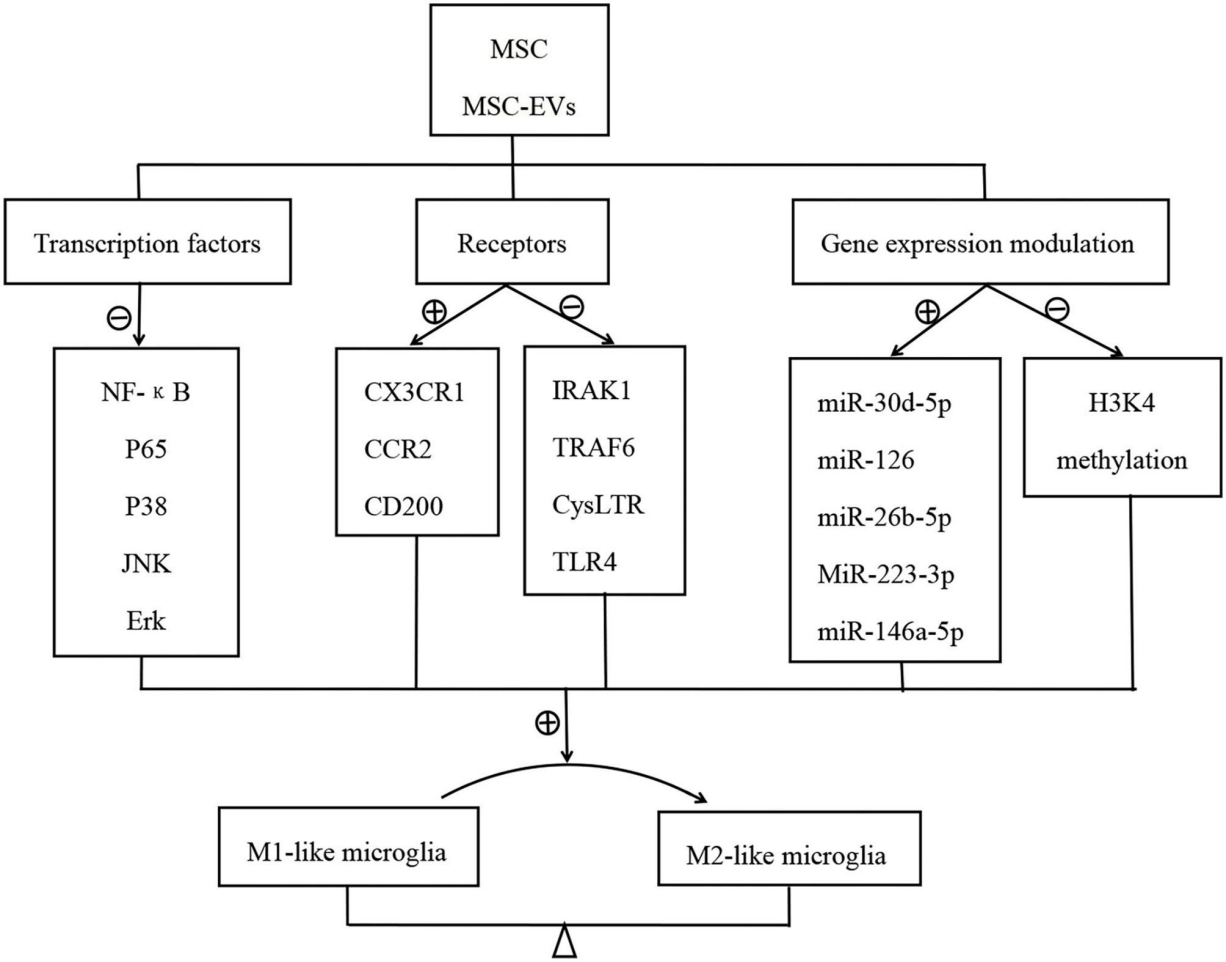
Mechanisms of MSC/MSC-EVs modulating the microglia activation.

Transcription factors are proteins that can regulate the transcriptional activity of genes by binding to DNA. NF-κB translocates from the cytoplasm into the nucleus in microglia following activation after ischemic stroke. Then, activate microglia were transformed into the M1 phenotype, followed by the production of inflammatory cytokines, resulting in secondary brain injury after stroke (98, 99). Neuroinflammatory responses can be attenuated in ischemic stroke *via* decreasing hypoxia-induced factor-1α following inhibition of activated NF-κB (p65) upregulation (100). MAPKs are a family of serine/threonine protein kinases, including JNK, ERK, and p38, which also play essential roles in the activation and polarization of microglia (101). MSC-derived soluble factor TSG-6 decreases NF-κB activity and the activation of MAPK signaling in LPS-induced microglia, in which the levels of expression of neuroinflammatory cytokines are reduced (62). B10 transplantation (a human MSC line) specifically decreases the NF-κB protein level in macrophage/microglia in the rat MCAO model (79). It is likely that MSC act on NF-κB signaling by inhibiting TLR2 and CD40 expression at the receptor level, increasing the inhibitory cytokine expression such as IL-4 and IL-10 at the activation level and inhibiting the expression of NF-κB itself (79) (see Figure 1).

Members of the signal transducer and activator of transcription (STAT) family of transcription factors have been demonstrated to play critical roles in cellular proliferation, immunity, and inflammation (102, 103). STAT3 can regulate macrophage/microglia polarization to anti-inflammatory phenotype (104, 105). However, some studies indicated that STAT3 could induce M1 microglia polarization in both an MCAO-induced and a bilateral common carotid arteries stenosis (BcaS)-induced model of ischemic stroke (106, 107). Consistently, hMSC reduced microglial STAT3 gene expression and activation of Y705 phosphorylated STAT3 following transplantation in an ouabain-induced brain ischemia rat model, which resulted in decreased microglia activation (108). The effect of STAT3 on microglia is likely contradictory, and further studies are required.

### Receptors

CX3CL1/CX3CR1 signaling is reported to regulate the microglia activity. It has been demonstrated that CX3CL1/CX3CR1 inhibits LPS-induced microglial activation and decreases expression of inflammatory factors in microglia such as NO, IL-6, and TNF-α *via* activating the PI3k/Akt pathway, which effectively protect the neuron (109, 110). The infusion of exogenous CX3CL1 reduced cerebral infarct size, neurological deficits, and caspase-3 activation in an MCAO mouse model (111). CX3CL1 expressed by B10 cells (human mesenchymal lines) inhibited cytokine-induced pro-inflammatory gene expression, including iNOS in a human microglia cell line *in vitro* (61). In addition, when transplanted in rat MCAO model, B10 cell decreased the accumulation of Iba-1^+^ microglia and inhibited pro-inflammatory gene expression in the core and ischemic border zone (IBZ) (61). In our study, BMSC intraventricular injection significantly increased the number of M2-type microglia (Iba^+^/CD206^+^ cells) and decreased the number of M1-type microglia (Iba^+^/TNFα^+^ cells) in the infarct region of MCAO rats, especially at 3 days and 7 days post transplantation (90). The above effects can be reversed *via* interfering with CX3CL1 administration, indicating that BMSC transplantation could shift microglia from pro-inflammatory type to anti-inflammatory in the infarct region of MCAO rats through triggering the secretion of CX3CL1 (90).

CCL2/CCR2 also plays an important regulatory role in post-stroke inflammation. Overexpressed CCL2 has been shown to increase macrophage infiltration and cerebral infarction area, indicating that CCL2 can promote inflammatory response and impede recovery post-stroke (112, 113). In a study, hUCMSC-derived exosome overexpressing CCR2 promoted M2 microglia/macrophage polarization possibly *via* competitively binding CCL2 to inhibit the excessive activation and M1 polarization of microglia/macrophages (86).

CD200, a member of the immunoglobulin superfamily, plays a critical role as a membrane glycoprotein in the maintenance of immune homeostasis. IL-4-treated hMSCs significantly reduced microglia secretion of IL-6 and IL-1β when co-cultured with LPS-activated microglia through CD200 expression on hMSCs (108). The immunomodulatory effect of hMSC CD200 expression was effectively inhibited by anti-CD200 antibodies (108). In another study, CD200 was more highly expressed under hypoxic conditions in human placenta amniotic membrane-derived mesenchymal stem cells (AMSCs) (63). When CD200 was silenced, the inhibitory effect of AMSC on pro-inflammatory cytokine expression in co-cultures with LPS-primed BV2 was decreased (63). Moreover, AMSC transplantation significantly decreased microglia activation in the boundary region in MCAO through upregulation of CD200 expression (63).

The receptor proteins IRAK1 and TRAF6 are widely distributed in the cytoplasm and nucleus of various cell types. They are largely involved in Toll-like receptor (TLR)-initiated pathways, which contribute to the expression of pro-inflammatory mediators (70). Overexpression of IRAK1 and TRAF6 can activate NF-κB (114). hUMSC-Exos administration decreased IBA-1^+^CD16^+^ (M1 type) and increased IBA-1^+^CD206^+^ (M2 type) microglia cells at 72 h post-stroke in the MCAO model (70). The immunomodulatory effect on microglia polarization may be mediated by miR-146a-5p, which binds to 3′UTR of the mRNAs encoding IRAK1 and TRAF6 and downregulates the inflammatory response (70).

Cysteinyl leukotrienes (CysLTs) are potent inflammatory mediators derived from the 5-lipoxygenase metabolites of arachidonic acid after cell necrosis (67). The action of CysLTs is mainly mediated by receptors of CysLT1 and CysLT2 (CysLT1R and CysLT2R), which are activated in various types of cells after brain injury (115). During cerebral ischemia, CysLTs receptors (mainly CysLT1R and CysLT2R) are upregulated in activated microglia (116). In a study, BMSC exosome overexpressing MiR-223-3p promoted M2 microglia transformation of M1 microglia when co-cultured with BV2 cells exposed to OGD *in vitro* through inhibiting CysLT2R (67). Concomitantly, pro-inflammatory factors such as IL-6 and IL-1β decreased and anti-inflammatory factors such as IL-10 increased (67).

Toll-like receptors (TLR) are signaling receptors in the innate immune system that can promote microglial activation and polarization (113, 117). Microglial activation was induced by TLR4 recognition of LPS on the cell surface after brain injury, followed by the production of pro-inflammatory factors *via* inhibiting the NF-κB pathway (118, 119). Targeted downregulation of TLR4 expression after ischemia-reperfusion injury can inhibit microglial activation and M1-type polarization, and reduce the release of pro-inflammatory factors (117). hUCMSCs-exos attenuated I/R-induced M1 microglia polarization and inflammatory response. The underlying mechanism maybe that exosomal miR-26b-5p could inhibit CH25H to inactivate the TLR pathway, resulting in the repression of M1 polarization of microglia (68).

### Modulation of gene expression

MicroRNAs (miRNAs) can regulate gene expression post-transcriptionally by combining with the 3'-UTR, coding sequence, or 5'UTR of target genes (120). They play key roles in the remodeling process under stroke conditions (121). MSC-derived exosomes are capable of transferring miRNAs to recipient cells and subsequently contribute to microglia polarization after stroke. Several studies elucidated the role of specific miRNAs in microglia polarization. ADSC-derived exosomes mediated the delivery of miR-30d-5p to the microglia and reversed OGD-induced and autophagy-mediated microglial polarization to M1 (64). The *in vivo* study also demonstrated that ADSC exosomal miR-30d-5p promoted M2 microglia/macrophage in the MCAO model by suppressing autophagy (64). IV administration of miR-126^+^ exosomes from ADSC post-stroke also inhibited microglial activation and the expression of inflammatory factors *in vivo* (65). hUCMSCs exosomal miR-26b-5p could repress M1 polarization of microglia by targeting CH25H to inactivate the TLR pathway after cerebral I/R (68). MiR-223-3p from BMSC exosome could promote M1 transformation to M2 microglia when co-cultured with BV2 cells exposed to OGD *in vitro* (67). As mentioned earlier, miR-146a-5p from hUMSC-Exos could promote the microglial shift from M1 to M2 after administration in the MCAO model *via* downregulation of IRAK1 and TRAF6 (70).

The rate of transcription of genes can be influenced by histone post-translational modifications (PTMs) at gene promoters and distal regulatory elements. Histone PTMs can exert negative and positive effects on transcription, including methylation (122). H3K4me1 increased dramatically in mice photothrombotic stroke models receiving LPS, in which CD16/32-positive microglia elevated and CD206-positive microglia decreased surrounding parainfarct region (87). When hUCMSC was administrated, microglial H3K4me1 modification was robustly alleviated, accompanied by a microglial shift from M1 toward M2 phenotype (87). Therefore, inhibiting H3K4 methylation maybe another putative mechanism for MSC to regulate microglia polarization.

## Conclusion

Immunity plays an important role in the pathophysiology of stroke. Microglia, as the first responsive immune cells to ischemia, is considered a double-edged sword in the progression of ischemic stroke. Pro-inflammatory microglia aggravate brain injury, while anti-inflammatory microglia are involved in repair after ischemic stroke. Modulating neuroinflammation *via* driving activated microglia to be neuroprotective might be novel therapeutics for ischemic stroke. An increasing number of evidence indicate that MSC and MSC-EVs could shift microglial phenotype from the pro-inflammatory M1-like state toward the anti-inflammatory M2-like phenotype. The underlying mechanisms involve several facets, including transcription factors, receptors, and gene expression. Further studies are required to elucidate the specific mechanisms of microglia polarization. Besides, cell source, dose, transplantation route, timing, and safety of MSC or MSC-EVs in ischemic stroke need to be further verified.

## Author contributions

LH, YY, and XX: acquisition of data and writing-original draft. XG and QZ: designing the review and writing–review and editing. All authors contributed to the article and approved the submitted version.
